# Supplementary material for: Multidrug-Resistant Staphylococcus sp. and Enterococcus sp. in Municipal and Hospital Wastewater: A Longitudinal Study
Source: Microorganisms. 2024 Mar 24;12(4):645. doi: 10.3390/microorganisms12040645 (PMC11051902; doi:10.3390/microorganisms12040645)
Supplement: Supplementary file 1 [file microorganisms-12-00645-s001.zip › microorganisms-2912624-supplementary.pdf]

Supplementary Material

Supplementary Figure S1. Antimicrobial susceptibility profiles of *Staphylococcus* sp. isolates present in community Wastewater Treatment Plant "Acapantzingo" (WWTP ACA) in Cuernavaca City.

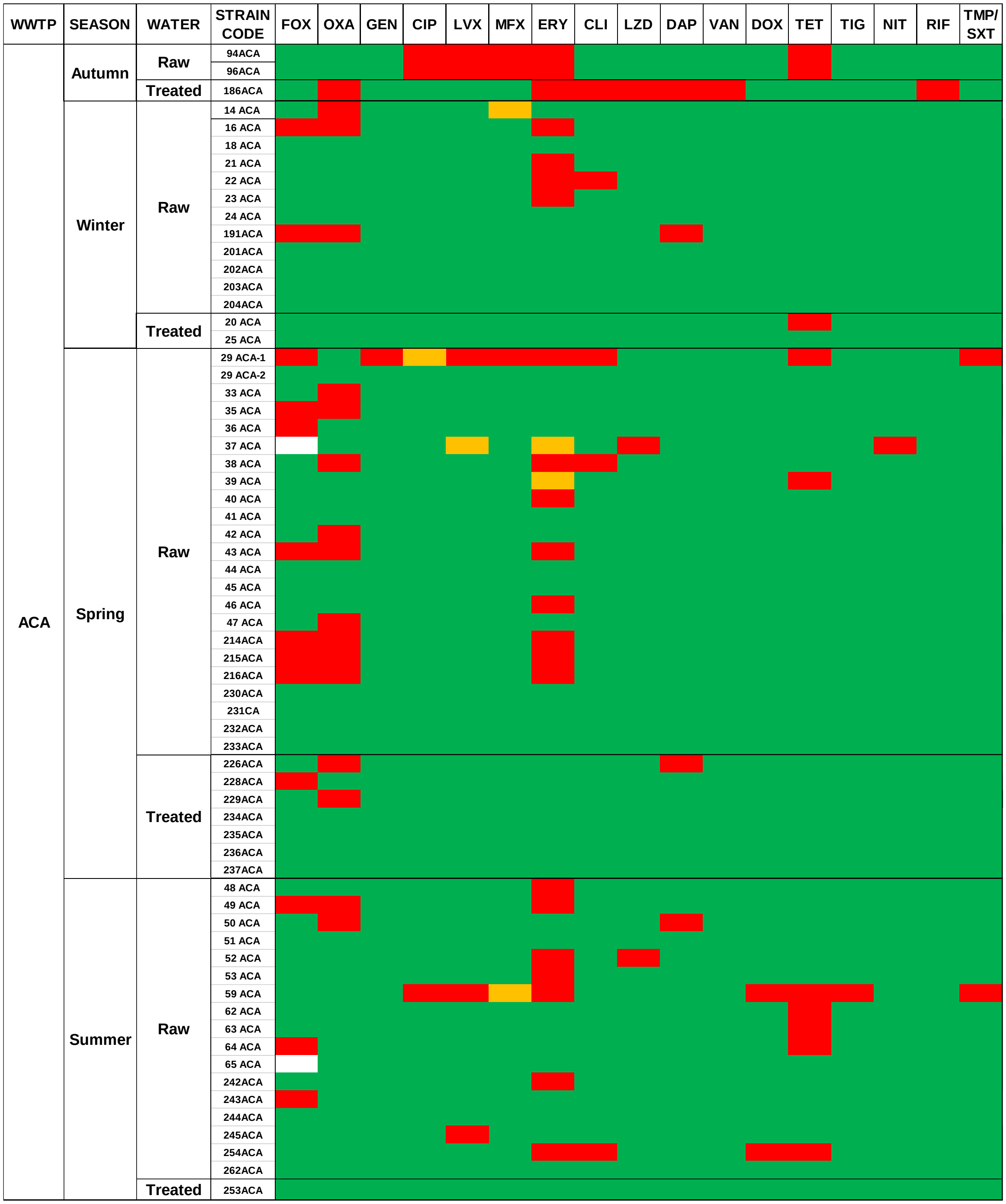

Green: Susceptible; Red: Resistant; Yellow: Intermediate; White: Not performed/Not determined. cefoxitin (FOX), oxacillin (OXA), gentamicin (GEN), ciprofloxacin (CIP), levofloxacin (LVX), moxifloxacin (MFX), erythromycin (ERY), clindamycin (CLI), linezolid (LZD), daptomycin (DAP), vancomycin (VAN), doxycycline (DOX), tetracycline (TET), tigecycline (TIG), nitrofurantoin (NIT), rifampin (RIF), trimethoprim-sulfamethoxazole (TMP/SXT).

Supplementary Figure S2. Antimicrobial susceptibility profiles of *Staphylococcus* sp. isolates present in community Wastewater Treatment Plant “Coyoacán” (WWTP COY) in Mexico City.

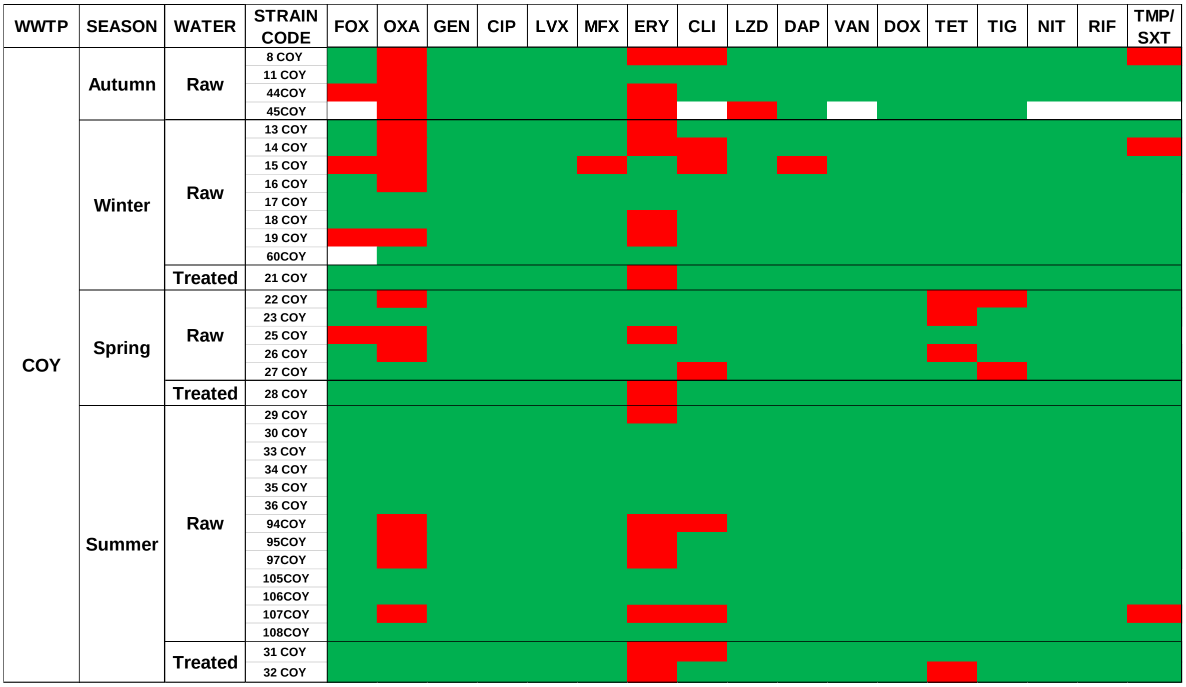

Green: Susceptible; Red: Resistant; Yellow: Intermediate; White: Not performed/Not determined. cefoxitin (FOX), oxacillin (OXA), gentamicin (GEN), ciprofloxacin (CIP), levofloxacin (LVX), moxifloxacin (MFX), erythromycin (ERY), clindamycin (CLI), linezolid (LZD), daptomycin (DAP), vancomycin (VAN), doxycycline (DOX), tetracycline (TET), tigecycline (TIG), nitrofurantoin (NIT), rifampin (RIF), trimethoprim-sulfamethoxazole (TMP/SXT).

Supplementary Figure S3. Antimicrobial susceptibility profiles of *Staphylococcus* sp. isolates present in hospital Wastewater Treatment Plant "Cancerología" (WWTP CAN) in Mexico City.

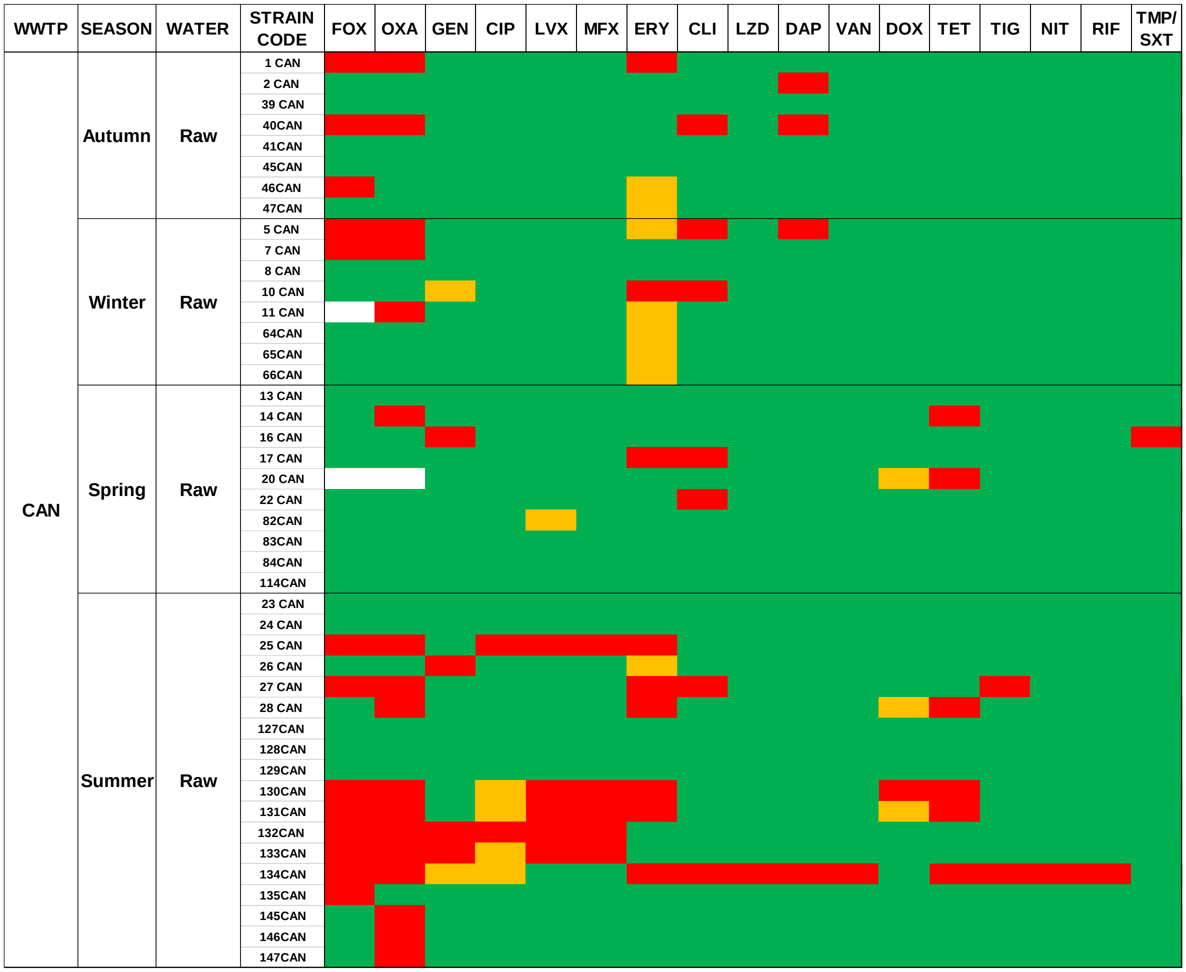

Green: Susceptible; Red: Resistant; Yellow: Intermediate; White: Not performed/Not determined. cefoxitin (FOX), oxacillin (OXA), gentamicin (GEN), ciprofloxacin (CIP), levofloxacin (LVX), moxifloxacin (MFX), erythromycin (ERY), clindamycin (CLI), linezolid (LZD), daptomycin (DAP), vancomycin (VAN), doxycycline (DOX), tetracycline (TET), tigecycline (TIG), nitrofurantoin (NIT), rifampin (RIF), trimethoprim-sulfamethoxazole (TMP/SXT).

Supplementary Figure S4. Antimicrobial susceptibility profiles of *Staphylococcus* sp. isolates present in hospital Wastewater Treatment Plant “Nutrición” (WWTP NUT) in Mexico City.

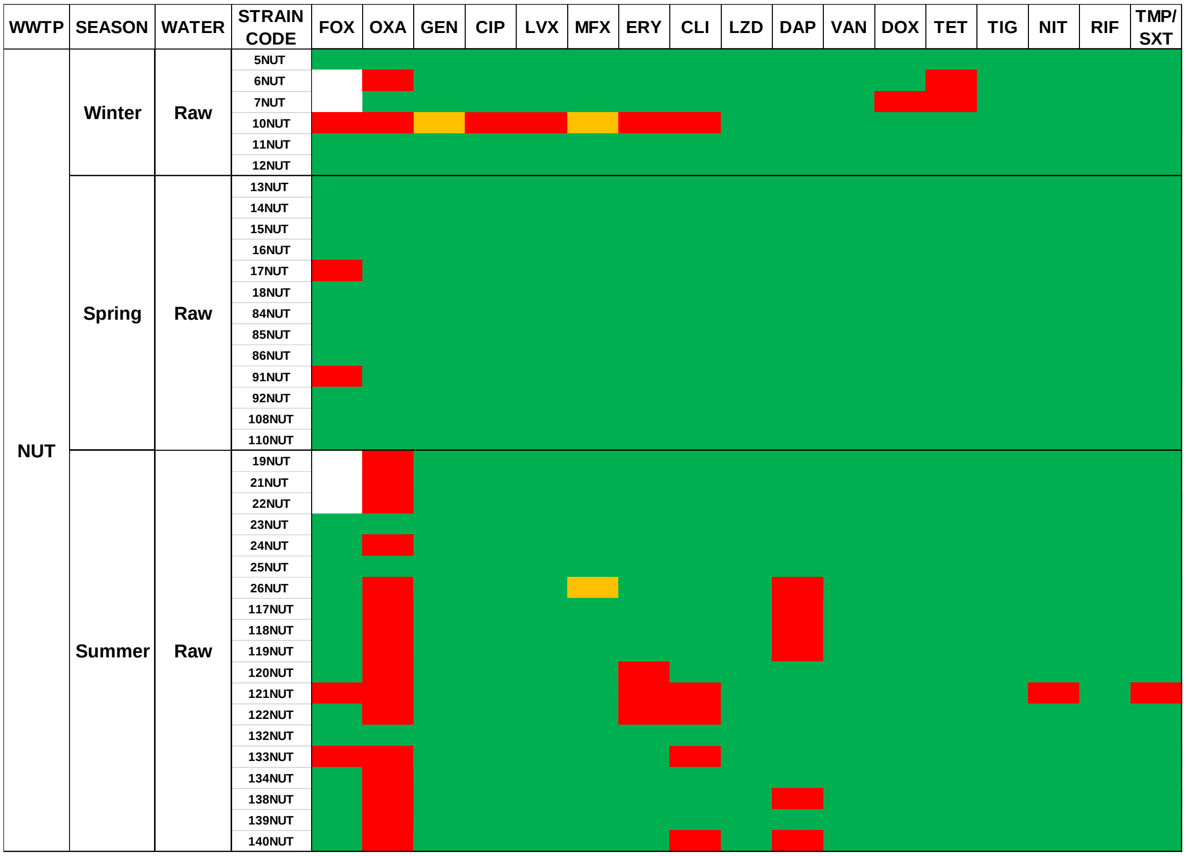

Green: Susceptible; Red: Resistant; Yellow: Intermediate; White: Not performed/Not determined. cefoxitin (FOX), oxacillin (OXA), gentamicin (GEN), ciprofloxacin (CIP), levofloxacin (LVX), moxifloxacin (MFX), erythromycin (ERY), clindamycin (CLI), linezolid (LZD), daptomycin (DAP), vancomycin (VAN), doxycycline (DOX), tetracycline (TET), tigecycline (TIG), nitrofurantoin (NIT), rifampin (RIF), trimethoprim-sulfamethoxazole (TMP/SXT).

Supplementary Figure S5. Antimicrobial susceptibility profiles of *Enterococcus* sp. isolates present in community Wastewater Treatment Plant “Acapantzingo” (WWTP ACA) in Cuernavaca City.

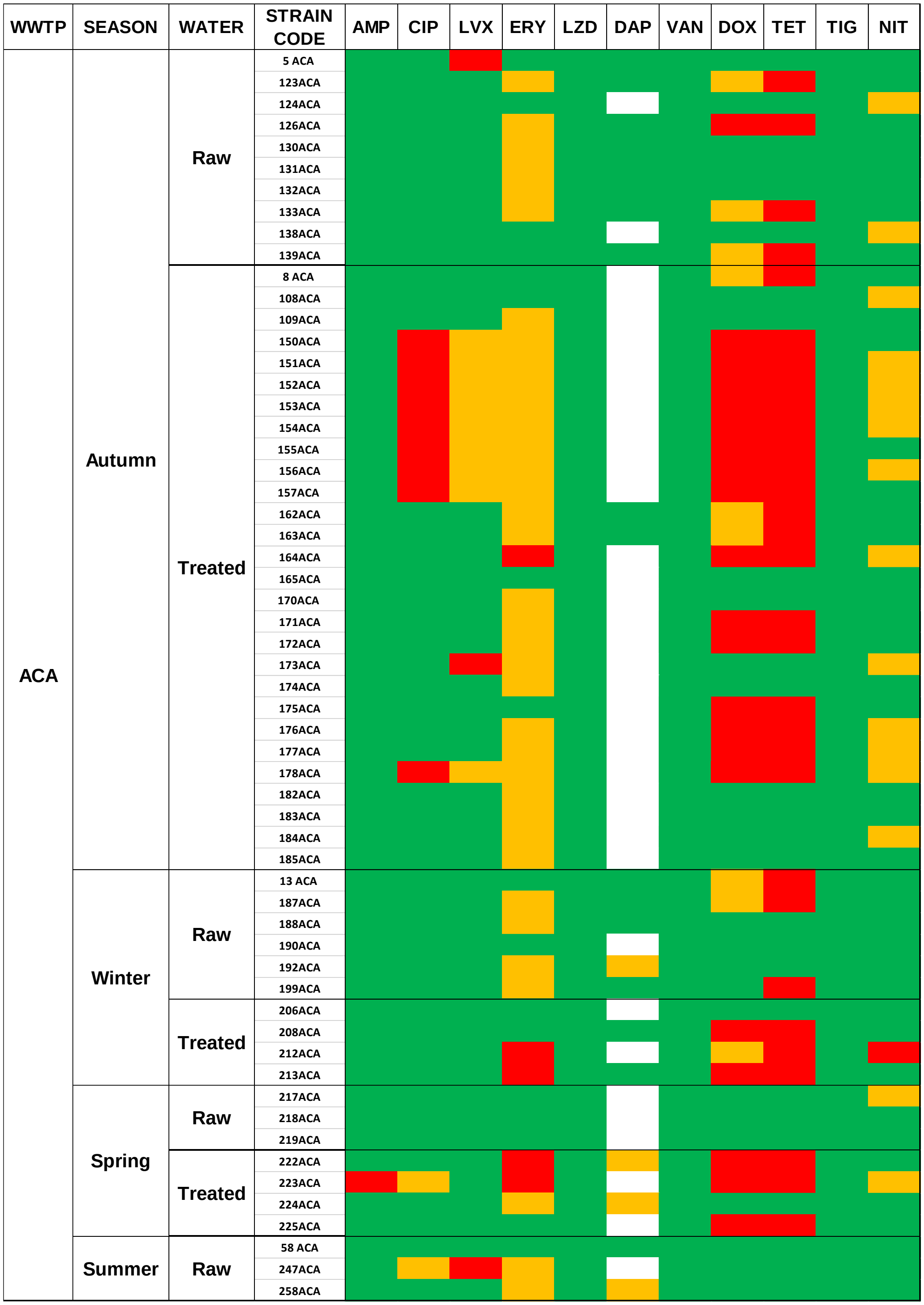

Green: Susceptible; Red: Resistant; Yellow: Intermediate; White: Not performed/Not determined. ampicillin (AMP), ciprofloxacin (CIP), levofloxacin (LVX), erythromycin (ERY), linezolid (LZD), daptomycin (DAP), vancomycin (VAN), doxycycline (DOX), tetracycline (TET), tigecycline (TIG), nitrofurantoin (NIT).

Supplementary Figure S6. Antimicrobial susceptibility profiles of *Enterococcus* sp. isolates present in community Wastewater Treatment Plant "Coyoacán" (WWTP COY) in Mexico City.

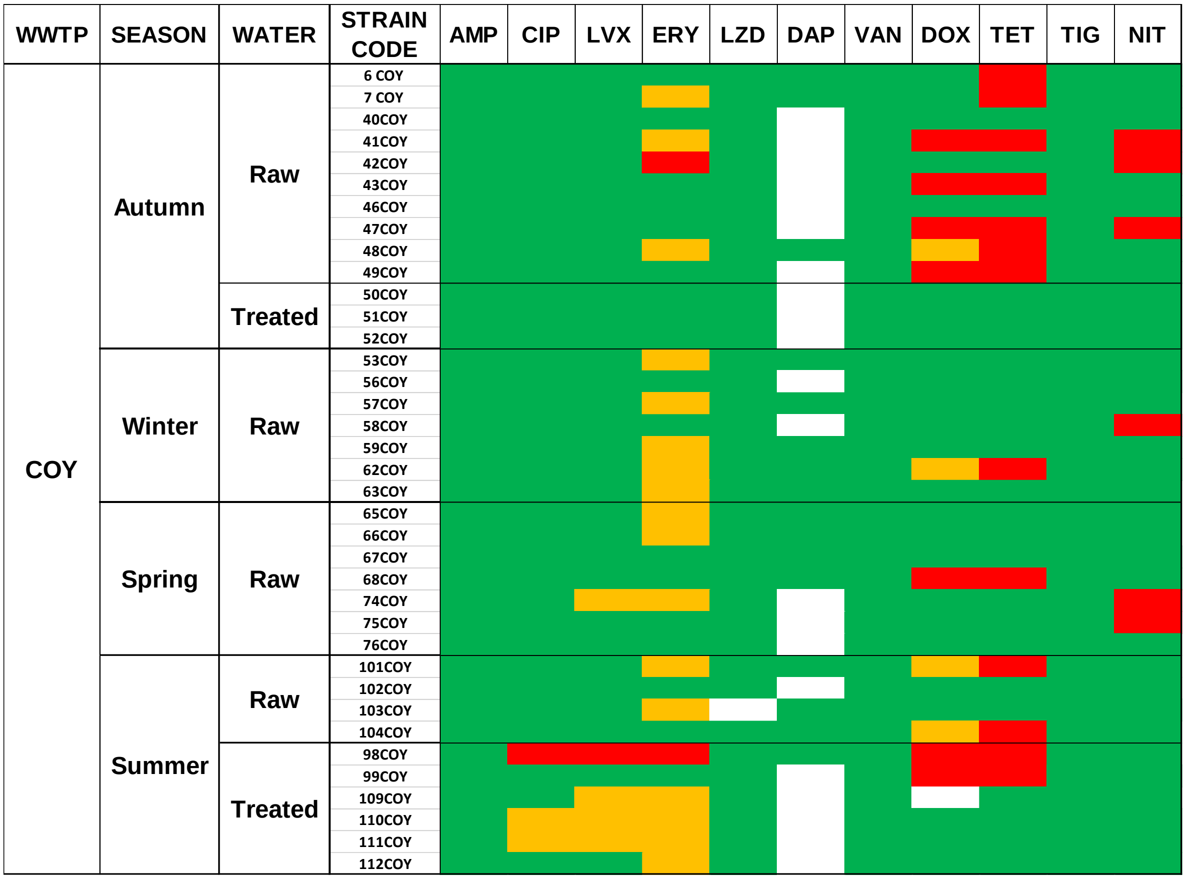

Green: Susceptible; Red: Resistant; Yellow: Intermediate; White: Not performed/Not determined. ampicillin (AMP), ciprofloxacin (CIP), levofloxacin (LVX), erythromycin (ERY), linezolid (LZD), daptomycin (DAP), vancomycin (VAN), doxycycline (DOX), tetracycline (TET), tigecycline (TIG), nitrofurantoin (NIT).

Supplementary Figure S7. Antimicrobial susceptibility profiles of *Enterococcus* sp. isolates present in hospital Wastewater Treatment Plant "Cancerología" (WWTP CAN) in Mexico City.

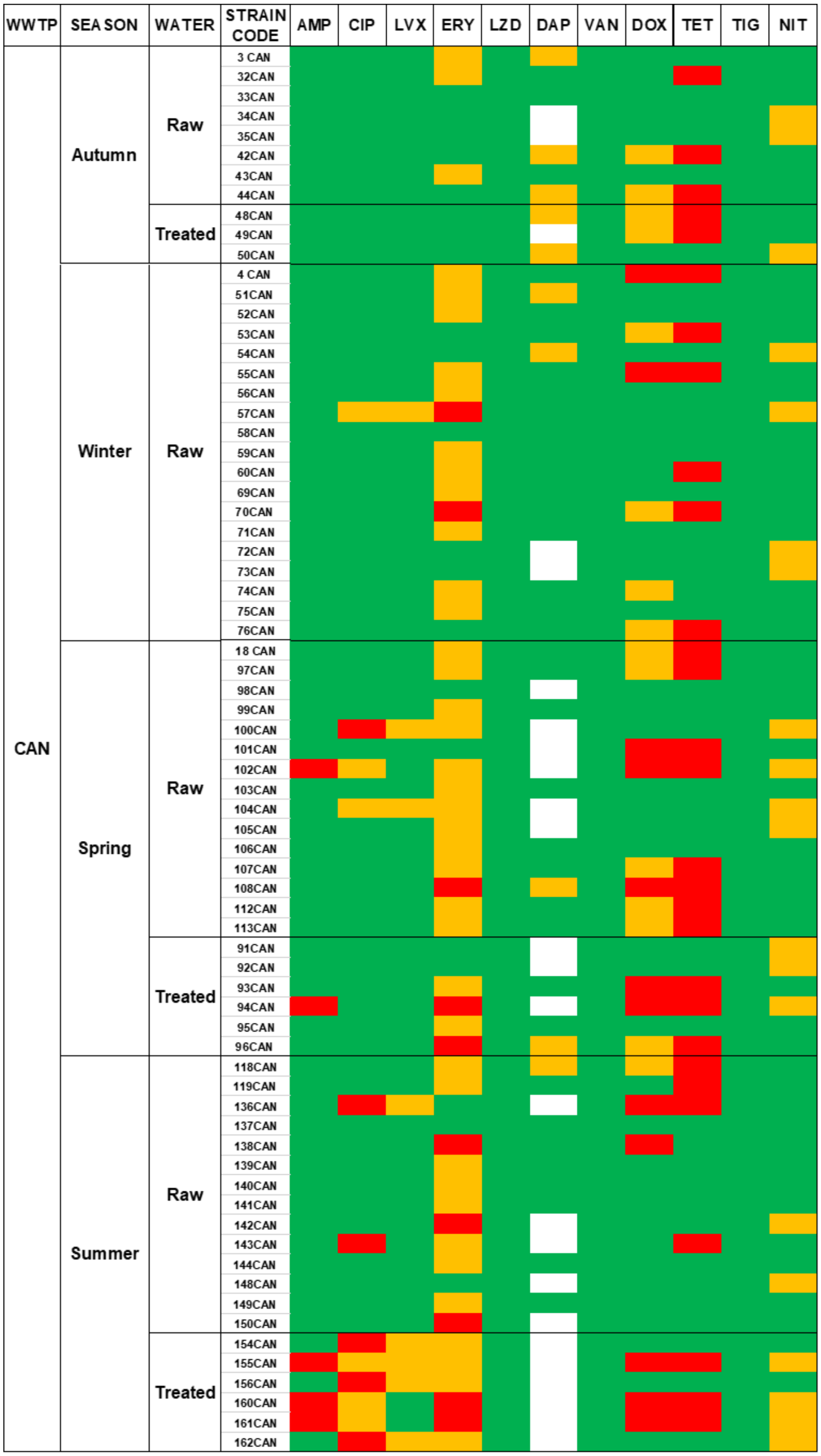

Green: Susceptible; Red: Resistant; Yellow: Intermediate; White: Not performed/Not determined. ampicillin (AMP), ciprofloxacin (CIP), levofloxacin (LVX), erythromycin (ERY), linezolid (LZD), daptomycin (DAP), vancomycin (VAN), doxycycline (DOX), tetracycline (TET), tigecycline (TIG), nitrofurantoin (NIT).

Supplementary Figure S8. Antimicrobial susceptibility profiles of *Enterococcus* sp. isolates present in hospital Wastewater Treatment Plant "Nutrición" (WWTP NUT) in Mexico City.

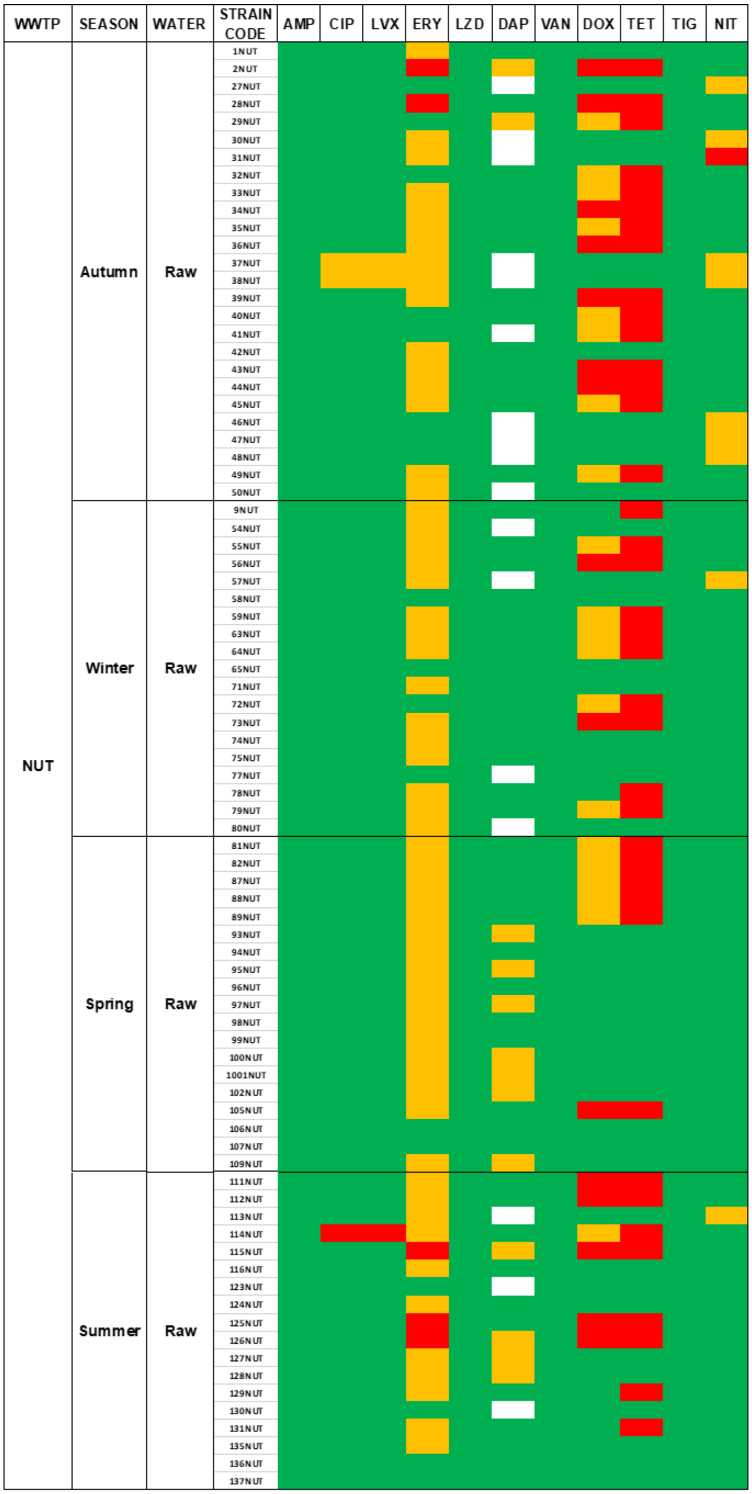

Green: Susceptible; Red: Resistant; Yellow: Intermediate; White: Not performed/Not determined. ampicillin (AMP), ciprofloxacin (CIP), levofloxacin (LVX), erythromycin (ERY), linezolid (LZD), daptomycin (DAP), vancomycin (VAN), doxycycline (DOX), tetracycline (TET), tigecycline (TIG), nitrofurantoin (NIT).
